# The impact of economic recession on the health of migrant fathers over time: results from the Growing up in Ireland longitudinal study

**DOI:** 10.1186/s12889-022-12596-0

**Published:** 2022-01-24

**Authors:** Nazmy Villarroel, Anne MacFarlane, Maria Roura, Alphonse Basogomba, Colette Bradley, Joseph W. LeMaster, Ailish Hannigan

**Affiliations:** 1 Department of Sociological Studies, The University of Sheffield, Sheffield, UK; 2 School of Medicine, University of Limerick, Limerick, Ireland; 3 Health Research Institute, University of Limerick, Limerick, Ireland; 4 School of Public Health, University College Cork, Cork, Ireland; 5 Intercultural and Diversity Education Centre - Ireland (IDEC-Ireland), Ennis, Co. Clare, Ireland; 6 Shannon Family Resource Centre, Shannon, Co. Clare, Ireland; 7 Department of Family Medicine and Community Health, University of Kansas School of Medicine, Kansas, USA

**Keywords:** Health status, Depression, Migrant men, Ethnicity, Cohort, Attrition

## Abstract

**Background:**

The relationship between economic conditions and health can depend on both the health outcome measured and the composition of the population. Analysis of outcomes by both ethnicity and country of birth has been recommended. The aim of our study is to explore the impact of recession on self-rated health and depression of migrant fathers in Ireland over time, considering both ethnicity and country of birth.

**Methods:**

Longitudinal data from waves of a population-representative cohort study (Growing up in Ireland, 2008–2013) was used with Wave 1 collected before the recession and Wave 2 collecting information on how the recession affected families. Socio-demographic variables, self-rated health and depression were compared across three groups of fathers classified by self-identified ethnicity and country of birth: White Irish (*n* = 5628), Other White European (EU-10) (*n* = 431), and Black African (*n* = 192) using chi-square tests and logistic regression models. Rates of follow-up were compared across groups at Wave 3.

**Results:**

Prior to the recession, the rate of employment was lowest for African fathers (51% vs 81% for EU-10 fathers and 92% for Irish fathers, *p* < 0.001). At Wave 2, African families were more likely to have experienced a very significant effect of the recession (40.1% compared to 22.4% for families from EU-10 and 21.3% for Irish families, *p* < 0.001). However, the impact of the recession on depression and self-rated health was only found in Irish fathers. By Wave 3, rates of follow-up were lower for migrant fathers, particularly for EU-10 fathers.

**Conclusions:**

Understanding the relationship between economic conditions and health is complex and may be related to multiple dimensions of socio-economic advantage and disadvantage. African families were already more likely to be disadvantaged prior to the recession and that pattern persisted during the recession. Further research on attrition rates of migrants in population cohort studies is needed and the development of effective strategies for recruitment, follow-up and analysis.

## Background

Economic conditions have significant effects on physical and mental health. Improvements in physical health are linked to economic growth through better nutrition, public health infrastructure and medical technology [1]. Poor mental wellbeing, increased rates of common mental disorders, and suicidal behaviours are linked to economic recession through the impact of job insecurity, unemployment, financial strain and debt [2, 3]. The relationship between economic conditions and health is, however, context dependent, can vary over time, and depend on both the health outcome measured and the composition of the population. Differences in the impact of economic conditions may be related to multiple dimensions of socio-economic advantage and disadvantage, such as age, gender, socio-economic position, level of education, family structure, migration status and ethnicity [3–5].

The ‘Great Recession’, a period of global economic decline during the late 2000s and early 2010s, is considered to be the longest period of economic decline since the Great Depression of the 1930s. Durbin, Page and Walby [5] reported that cuts to income and public services at the heart of austerity policies during the recession were disproportionately borne by those with intersecting disadvantages of poverty, disability, ethnicity and age. Raftery [4] reported that ethnic minority men and women in the UK fared worse in the labour market during the recession than the white UK born majority. The highest increase in unemployment for men was in Black men (African Caribbean/Black African/Black Other). However, there are conflicting reports in the literature on the impact of the recession on migrants. Simona-Moussa and Ravazzini [6], using household panel data, reported that the impact on migrants in Switzerland was seen in objective measures of quality of life such as income poverty and material deprivation but not in subjective measures such as wellbeing and satisfaction. Selective attrition in longitudinal studies of those most at risk of poorer outcomes has, however, been highlighted as a challenge for drawing conclusions over time for marginalized groups [6].

Ireland experienced a decade of rapid economic growth from the mid-1990s with the unemployment rate constant at about 4% from 2000 to 2007. The country also experienced a significant inflow of migrants during this time, particularly in the period after May 2004 when the European Union (EU) admitted ten new member states including Poland, Lithuania and Latvia. Ireland was one of the few countries to allow immediate access to its labour market to new members [7]. The Great Recession had a profound impact on the country with a loss of economic sovereignty in 2010. Austerity measures were characterized by increases in taxation and reductions in public expenditure including cuts to welfare and health care spending. Unemployment peaked at 15% in 2012 [8], and rates of unemployment were higher for non-Irish nationals compared to Irish nationals, particularly for migrant men who had worked in the construction industry.

Corcoran et al. [9] reported a significant negative impact of economic recession and austerity in Ireland on rates of suicide in men and rates of self-harm in both sexes. Reinhard et al. [10] reported that the recession negatively impacted the health of children in Ireland, particularly those who were socioeconomically vulnerable. No study, however, has reported on the impact of the recession on the health of migrant men in Ireland using longitudinal data and considering both ethnicity and country of birth. The experiences of migrant men has received less attention than migrant women in the literature [11], particularly migrant fathers who play a critical role in supporting their families adjust to life in a new country and can feel pressure to financially provide for their family [12].

The aim of our study, therefore, is to examine whether the impact of the recession on self-rated health and depression of fathers varied by country of birth and ethnicity. We use longitudinal data from two waves of a population-representative cohort study (Growing up in Ireland, 2008–2013) with Wave 1 collected before the recession and Wave 2 collecting detailed information on how the recession affected families. Given the potential impact of the recession on long-term follow-up in migrant fathers, we also explore rates of follow-up across groups at Wave 3.We focus on three groups of fathers (categorised by country of birth and ethnicity) and compare self-rated health and depression across groups.

## Methods

This research was conducted as part of a mixed-methods, interdisciplinary participatory health research study, described in the study protocol [13]. A Steering Group composed of different stakeholders including community members oversaw the conduct of this research. This paper focuses on the quantitative component, specifically analysis of an existing dataset to generate evidence about ethnic minority and majority health in Ireland. The discussions of the Steering Group guided the selection of groups and health outcomes to compare and interpretation of the findings in context. The dataset is described next, followed by a description of how the data for this study was analysed.

### Growing up in Ireland Study

The National Longitudinal Study of Children Growing up in Ireland (GUI) is a government funded study that focuses on the developmental trajectories of children in Ireland. Parents of all children in Ireland receive a monthly payment known as Child Benefit from birth to the age of 16. It must be applied for within 12 months of the birth of the child or the family coming to live in the country. The payment is universal, regardless of income level, as long as the parent is habitually resident in the country. Asylum seekers are not considered habitually resident until protection has been granted and therefore are not eligible for Child Benefit. A total of 73,662 infants were recorded on the Child Benefit Register for the calendar year 2008. A stratified random sample of 16,136 infants born between December 2007 and May 2008 was selected from the Child Benefit Register and their parents invited to take part in the Infant Cohort of GUI when their child was 9 months of age. Of those invited, 11,134 agreed to participate (response rate of 64%) with interviewing for Wave 1 taking place in 2008/9 [14]. The second wave of data was collected in 2011 when the child was aged 3 years and the third wave was collected in 2013 when the child was aged 5 years.

A trained fieldworker carried out a computer-assisted personal interview in the family home, interviewing the Primary Caregiver, defined as the person who provides most care to the infant. If the Primary Caregiver had a spouse or partner living in the household, this person was assigned to be the Secondary Caregiver and was also interviewed. Of the 11,134 infants in the cohort, 8632 (78%) had both Primary and Secondary Caregivers interviewed. Almost all Secondary Caregivers were male and the father of the child (*n* = 8597), and are referred to hereafter as ‘fathers’.^1^

Fathers were asked to give their country of birth and also to self-identify their ethnic or cultural background into one of the following categories: White Irish, Irish Traveller, Any other white background, Black African, Any other Black background, Chinese, Any other Asian background, or Other (including mixed background) with a free text option to specify. These categories are the categories used in the Census of Population in Ireland since 2006 and were used in GUI to facilitate comparison of participant characteristics with the population.

**Fig. 1:**
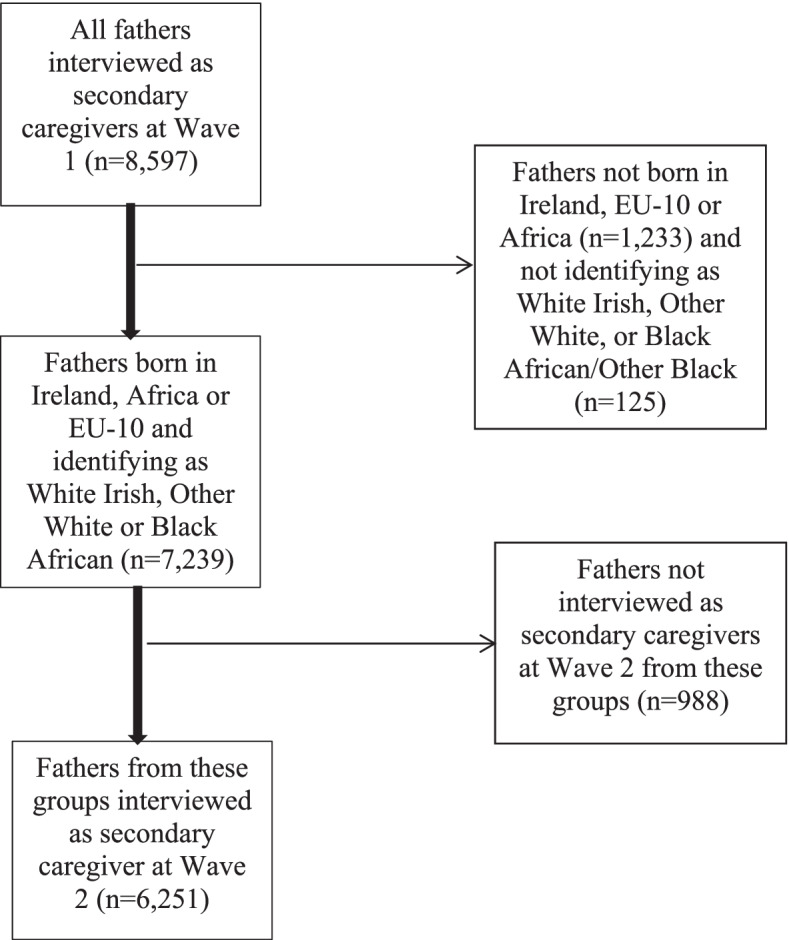
Study flow chart of sample included in analysis

Our sample for analysis is fathers who were interviewed at Wave 1 and 2 as a secondary caregiver (see Fig. 1 for a study flow chart) and who self-identified in one of three groups below.

### Groups by ethnicity and migration status

- Fathers who were born in Ireland and who self-identified as belonging to the majority ethnic group ‘White Irish’ (*n* = 5628) –hereafter called Irish fathers
- Fathers who were born in one of the ten countries who joined the EU in 2004 (Cyprus, Czech Republic, Estonia, Hungary, Latvia, Lithuania, Malta, Poland, Slovakia, and Slovenia) and who self-identified as belonging to the largest minority ethnic group ‘Any other White background’ (*n* = 431) – hereafter called EU-10 fathers
- Fathers who were born in an African country and who self-identified as belonging to the minority ethnic groups ‘African’ or ‘Any other Black background’ (*n* = 192) – hereafter called African fathers

The two comparison groups to the majority Irish-born group were chosen to reflect diversity in employment patterns, language, freedom of movement across the EU and experience of discrimination. Men from the ten EU new member states had the highest employment rate (93%) during the period of economic growth in Ireland compared to 59% for African men but also experienced the most significant drop in employment (rate of employment of 68% in 2010 compared to 55% for African men) [15]. McGinnitty et al. [16] reported that Black Africans are more likely to experience discrimination and harassment, both in employment and in public spaces, than other ethnic minorities in Ireland.

The three groups of fathers in our study represent the majority (84%) of all fathers interviewed at Wave 1 of GUI. We excluded fathers born in the United Kingdom (*n* = 636, 7%) from our study. These are generally considered separately to other migrants in Ireland because of the long history of migration and common travel area between these countries, shared language and a significant proportion of UK-born in Ireland having Irish citizenship [17].

We also describe other variables in GUI related to ethnicity and migration for the three groups in our study including having Irish citizenship (Yes/No); language competence which was measured by the question: “Can you usually read and fill out forms you might have to deal with in English?” (Yes/No); religion (Catholic, Other Christian, Muslim, Other, None); and for those not born in Ireland, time spent living in Ireland (< 5 years, 6–10 years and more than 10 years).

The overall follow up rate from Wave 1 to Wave 2 was 86%, ranging from 71% for EU-10 fathers to 79% for African fathers and 88% for Irish fathers.

### Socio-demographic variables

Socio-demographic variables included highest level of education (No formal education/primary, secondary, technical qualification, degree or higher), whether the family were entirely dependent on social welfare for household income, family’s socio-economic position categorized in five groups (professional/managerial, non-manual/skilled manual, semi-skilled/unskilled, others and never worked) and employment status of the father (Employed, Student/training scheme, looking for work, other).

### Health outcomes

- Self-rated health which was assessed by asking respondents: In general, how would you say your current health is?’ with the possible choices being ‘Excellent’, ‘Very good’, ‘Good’, ‘Fair’ or ‘Poor’. We analysed self-rated health as a dichotomous measure of self-rated health with ‘Excellent’, ‘Very good’ or ‘Good’ coded as 0, and ‘Fair’ or ‘Poor’ coded as 1.
- The eight-item Center for Epidemiologic Studies Depression Scale (CESD), which was designed to screen for depressive symptomatology among a population during the 7 days preceding assessment. Scores on the CESD can range from 0 to 24, with higher scores indicative of a higher incidence of both the presence and duration of symptoms. A threshold score of ≥7 on the total scale score is recommended to classify respondents as depressed. The CESD demonstrated good internal reliability among fathers in GUI (Cronbach’s α = 0.81).

### Measures of recession impact

The second wave of GUI asked mothers to rate the extent to which the recession had had an impact on their household, on a 4-point scale (‘no effect’, ‘small effect’, ‘significant effect’, or ‘very significant effect’). Those who reported at least a small effect were asked more detailed questions about how the recession had affected their household. Possible responses were 1) mother’s job loss, 2) father’s job loss, 3) a reduction in working hours for either partner, 4) a reduction in wages for either partner, 5) a reduction in social welfare benefits, 6) the household falling behind on rent or mortgage payments, 7) the household falling behind on utility bills, and 8) the household being unable to afford or having to cut back on basic necessities, such as food and clothing.

### Statistical Analysis

Categorical data was summarised using frequencies and percentages. Differences in categorical variables related to ethnicity and migration for African and EU-10 fathers were tested using the Z-test for proportions. The chi-square test was used to test for associations between socio-demographic variables and impact of the recession across three groups (Irish, EU-10, African). A 5% level of significance was used for all tests. The strength of the association between categorical variables was measured using Cramer’s V with 0.1 considered a small effect, 0.3 a moderate effect and > 0.3 a large effect. Binary logistic regression models were fitted to predict depression status and self-rated health at Wave 2, adjusting for group (Model 1), age (Model 2) and level of education, whether the family was entirely dependent on social welfare for household income at Wave 1 and the impact of the recession at Wave 2 (Model 3). Odds ratios (OR) with 95% confidence intervals (CI) are reported. Goodness of fit was assessed using the Hosmer-Lemeshow chi-squared test. Given the potential for multiple interactions with group [18], models were also fitted separately to each group. The Statistical Package for the Social Sciences statistical software package version 24.0 (SPSS, Inc., Chicago, IL, USA) was used for the statistical analysis.

## Results

Table 1 summarises variables related to migration and ethnicity by group. When comparing these variables across the two migrant groups, the rate of English language competency was lower in EU-10 fathers compared to African fathers (77% vs. 97%, *p* < 0.001). EU-10 fathers were also more likely to be recently arrived in Ireland (≤ 5 years) compared to African fathers (84% vs. 44%, *p* < 0.001). Rates of Irish citizenship were low in both EU-10 fathers and African fathers (3% vs. 13%, *p* < 0.001).

**Table 1:** Comparing variables related to migration and ethnicity across groups (Irish, EU-10, African)

Group	Irish (***n*** = 5628, 90%)	EU-10 (***n*** = 431, 7%)	African (***n*** = 192, 3%)
**Citizenship**	5626 (99%)	14 (3%)	25 (13%)
**English language competency**	5509 (98%)	331 (77%)	187 (97%)
**Religion**			
Roman Catholic	5018 (89%)	277 (64%)	41 (21%)
Other Christian	203 (4%)	101 (23%)	108 (56%)
Muslim	–	–	39 (21%)
None	399 (7%)	47 (11%)	–
**Length of time in Ireland**			
≤ 5 years		359 (84%)	85 (44%)
6–10 years		67 (16%)	100 (52%)
> 10 years		–	7 (4%)

Table 2 summarises socio-demographic variables measured at Wave 1, prior to the recession, across the three groups. EU-10 fathers were younger than Irish or African fathers with 46% under the age of 30 years compared to 13% for Irish fathers and 8% for African fathers. The rate of employment (employee, self-employed or farmer) was lowest for African fathers (51% vs 81% for EU-10 fathers and 92% for Irish fathers, *p* < 0.001). As a consequence, more African families were dependent on social welfare with a third of these families having no other household income. African fathers were, however, more likely to have at least a degree level qualification (45% vs. 22% for EU-10 fathers and 32% for Irish fathers, p < 0.001). The heterogeneity within African fathers is evident in socio-economic position with a higher proportion in the professional/managerial category compared to EU-10 fathers but also the highest proportion of never worked across groups. Of the African fathers not currently in paid employment outside the home (*n* = 66), 23 (35%) stated that they were unable to work because of the lack of a work permit/seeking asylum.

**Table 2:** Comparing socio-demographic variables measured before the recession across groups (Irish, EU-10, African)

Group	Irish (***n*** = 5628)	EU-10 (***n*** = 431)	African (***n*** = 192)	***P***-value (Cramer’s V)
**Age group**				
< 30 years	726 (13%)	197 (46%)	15 (8%)	< 0.001 (0.18)
30-34 years	1862 (33%)	159 (37%)	49 (26%)	
35-39 years	1962 (35%)	55 (13%)	74 (38%)	
≥ 40 years	1078 (19%)	20 (5%)	54 (28%)	
**Current employment status**				
Employed	5172 (92%)	350 (82%)	98 (51%)	< 0.001 (0.21)
Student/training scheme	38 (1%)	–	27 (14%)	
Looking for work	341 (6%)	69 (16%)	50 (26%)	
Other	76 (1%)	5 (1%)	16 (8%)	
**All household income derived from social welfare**	197 (4%)	29 (7%)	62 (33%)	< 0.001 (0.24)
**Highest level of education**				
Primary	151 (3%)	7 (2%)	5 (3%)	< 0.001 (0.10)
Secondary	1861 (33%)	89 (21%)	51 (27%)	
Technical/non degree	1802 (32%)	239 (55%)	50 (26%)	
Degree or higher	1814 (32%)	94 (22%)	86 (45%)	
**Socio-economic position**				
Professional/managerial	3518 (62%)	67 (15%)	64 (33%)	< 0.001 (0.30)
Non-manual/skilled manual	1686 (30%)	218 (51%)	39 (20%)	
Semi-skilled/unskilled	328 (6%)	130 (30%)	39 (20%)	
Others	–	11 (3%)	–	
Never worked	93 (2%)	5 (1%)	50 (26%)	

Table 3 summarises the extent of the effect of the recession on the three groups, measured at Wave 2. African families were more likely to have experienced a very significant effect (40.1% compared to 22.4% for families from EU-10 and 21.3% for Irish families, *p* < 0.001). Of those who experienced some effect of the recession, fathers from EU-10 were more likely to have lost their jobs while African families were more likely to have social welfare payments reduced compared to Irish families. African families were also more likely to have had to cut back on the basics and be behind in rent and utility bills (Table 3).

**Table 3:** Comparing the effects of the recession across groups (Irish, EU-10, African)

Group	Irish (***n*** = 5628)	EU-10 (***n*** = 431)	African (***n*** = 192)
**Extent of the effect of the recession**			
Very significant	1196 (21%)	95 (22%)	77 (40%)
Significant	2197 (39%)	128 (30%)	66 (34%)
Small	1911 (34%)	152 (36%)	35 (18%)
None	324 (6%)	50 (12%)	14 (7%)
Of those who experienced at least some effect of the recession:			
**Father lost job/made redundant**	1060 (20%)	132 (35%)	42 (24%)
**Mother lost job/made redundant**	594 (11%)	56 (15%)	19 (11%)
**Social welfare reduced**	2387 (45%)	219 (58%)	120 (67%)
**Working hours reduced**	1288 (23%)	131 (35%)	48 (27%)
**Cannot afford or had to cut back on basics**	1388 (26%)	97 (26%)	84 (47%)
**Behind with rent or mortgage payments**	324 (6%)	29 (8%)	30 (17%)
**Behind with utility bills**	459 (9%)	39 (10%)	68 (38%)

The proportion of fathers rating their health as poor/fair was low across all three groups and there was no statistically significant association between rating health as poor/fair and group at either Wave 1 or Wave 2 (Table 4). Similarly, there was no statistically significant association between depression and group at Wave 2.

**Table 4:** Comparing health outcomes across groups (Irish, EU-10, African) pre- and post- recession ^a^

Group	White Irish and born in Ireland (***n*** = 5628)	Any other White and born in EU-10 (***n*** = 431)	Black African or Other Black and born in Africa (***n*** = 192)	***P***-value
**Health status**				
Fair/Poor				
Wave 1	276 (5%)	17 (4%)	6 (3%)	0.37
Wave 2	194 (4%)	15 (4%)	9 (5%)	0.55
**Depression**				
Wave 1	176 (3%)	12 (3%)	12 (6%)	0.045
Wave 2	223 (4%)	11 (3%)	12 (7%)	0.08

^a^Missing data for some participants, valid percentages given

In a regression model for depression at Wave 2, the recession having an impact on the family was associated with increased odds of depression (OR 5.69, 95% CI 2.06 to 15.66 for a very significant effect of the recession compared to no effect). In separate regression analyses for each group (Irish, EU-10, African), the impact of the recession on depression was seen in Irish fathers but not EU-10 or African fathers (Table 6). The family being completely dependent on social welfare at Wave 1 was also associated with increased odds of depression at Wave 2 (OR 2.07, 95% CI 1.27, 3.38).

In a regression model for self-rated health at Wave 2, only lower levels of education and the family being completely dependent on social welfare at Wave 1 were associated with increased odds of self-rating health as poor/fair (Table 5). The impact of the recession on self-rated health was only seen in Irish fathers but not EU-10 or African fathers (Table 6).

**Table 5:** Binary logistic regression with health outcomes of self-rated health (poor/fair) and depression after the recession (*n* = 6251)

	Self-rated health (poor/fair) Odds ratio (95% CI)	Depression Odds ratio (95% CI)
**Model 1**		
**Group**		
White Irish	Reference	Reference
EU-10	1.05 (0.61, 1.79)	0.69 (0.38, 1.28)
Black African	1.46 (0.74, 2.90)	1.75 (0.96, 3.20)
**Model 2**		
**Group**		
White Irish	Reference	Reference
EU-10	1.11 (0.64, 1.95)	0.63 (0.33, 1.17)
Black African	1.39 (0.70, 2.78)	1.80 (0.98, 3.29)
**Age group**		
< 30 years	Reference	Reference
30–34 years	0.72 (0.47, 1.13)	0.87 (0.59, 1.28)
35–39 years	0.89 (0.57, 1.37)	0.79 (0.53, 1.17)
≥ 40 years	1.52 (0.97, 2.37)	0.69 (0.43, 1.08)
**Model 3**		
**Group**		
White Irish	Reference	Reference
EU-10	1.16 (0.64, 2.11)	0.53 (0.26, 1.10)
Black African	0.95 (0.44, 2.03)	1.29 (0.68, 2.46)
**Age group**		
< 30 years	Reference	Reference
30–34 years	0.90 (0.57, 1.41)	1.04 (0.69, 1.57)
35–39 years	1.17 (0.75, 1.83)	0.98 (0.65, 1.48)
≥ 40 years	1.72 (1.09, 2.70)	0.79 (0.49, 1.27)
**Highest level of education**		
Primary	6.79 (3.75, 12.30)	1.34 (0.63, 2.83)
Secondary	2.26 (1.51, 3.38)	1.18 (0.84, 1.66)
Technical/non-degree	1.58 (1.04, 2.41)	1.03 (0.73, 1.46)
Degree/Post-grad	Reference	Reference
**Dependent on social welfare for all household income**		
No	Reference	Reference
Yes	1.99 (1.23, 3.20)	2.07 (1.27, 3.38)
**Impact of the recession on family at Wave 2**		
None	Reference	Reference
Small effect	1.02 (0.48, 2.18)	2.96 (1.07, 8.20)
Significant effect	1.54 (0.73, 3.22)	3.39 (1.24, 9.33)
Very significant effect	2.03 (0.96, 4.30)	5.69 (2.06, 15.66)

**Table 6:** Binary logistic regression with health outcomes of self-rated health (poor/fair) and depression after the recession by group (Irish, EU-10, African)

	Irish (***n*** = 5628)	EU-10 (***n*** = 431)	African (***n*** = 192)
**Depression (yes)**			
**Impact of the recession on the family at Wave 2**			
None	Reference	Reference	Reference
Small	5.37 (1.31, 22.08)	0.23 (0.04, 1.41)	0.36 (0.04, 2.83)
Significant	6.36 (1.56, 25. 97)	0.27 (0.04, 1.66)	0.21 (0.03, 1.64)
Very significant	10.88 (2.66, 44.55)	0.75 (0.16, 3.53)	0.56 (0.10, 3.14)
**Self-rated health (poor/fair)**			
**Impact of the recession on the family at Wave 2**			
None	Reference	Reference	Reference
Small	1.46 (0.57, 3.71)	0.58 (0.13, 2.53)	0.38 (0.02, 6.48)
Significant	2.49 (1.00, 6.18)	0.40 (0.08, 2.07)	0.21 (0.01, 3.61)
Very significant	3.58 (1.43, 8.98)	0.71 (0.15, 3.33)	1.20 (0.13, 10.89)

### Long-term follow-up

Our sample for analysis included fathers who were interviewed as secondary caregivers at both Wave 1 and Wave 2. Given the potential impact of the recession on long-term follow-up in migrant fathers, we also explored rates of follow-up across groups at Wave 3. By Wave 3, only 57% of EU-10 fathers had participated in all three Waves compared to 68% of African fathers and 81% of Irish fathers.

## Discussion

We defined two distinct groups of migrant fathers by country of birth and ethnicity. Analysis of outcomes by both ethnicity and country of birth has been recommended to bring new perspectives to understanding health status by accounting for those born abroad who belong to the majority ethnic group [19]. This is particularly relevant in Ireland where a long history of emigration and returning emigrant families has resulted in many second-generation Irish living in Ireland and identifying as the majority ethnic group ‘White Irish’. Including these in a crude analysis of ‘foreign-born’ vs. ‘Irish-born’ can minimise differences in health outcomes across groups. The two distinct groups of migrant fathers in our study differed by country of birth and ethnicity but also other related variables such as length of stay in Ireland, English language competency and Irish citizenship. Disaggregating migration status by these variables as well as intersecting it with ethnicity can lead to a better understanding of the effect migration status has on health [20].

We also identified differences in socio-demographic variables across groups. African families were more likely to be dependent on social welfare and some of this was accounted for by structural barriers to employment. McGinnitty et al. [21] reported that those born in Africa have much lower employment and activity rates than other migrant groups in Ireland. This may be influenced by time spent in the asylum system and not in the labour market for those who are seeking protection, and potentially also the experience of racism and discrimination in the Irish labour market. There was considerable heterogeneity, however, in level of education and socio-economic position within the group of African fathers in our study. This highlights the importance of an intersectionality approach when understanding health outcomes, reflecting the interaction between gender, country of birth, ethnicity, as well as social position and conditions at different stages of the migration journey and settlement [20, 22, 23].

The rate of employment in EU-10 fathers, with freedom of labour movement across Europe, was high and the majority were non-manual or skilled manual workers. A recent bibliometric analysis of global migration health research [24] highlighted the gap in research about migrant workers, accounting for just 6% of over 20,000 papers included in the analysis. Understanding the health of these young, mobile workers is important with some evidence of increased rates of occupational injuries in these workers in Ireland [25, 26]. The quality of work, i.e. the extent to which it fosters beneficial outcomes such as physical wellbeing for the employee, has been shown to be an important determinant of health with some migrant workers particularly vulnerable to a lower quality of work [27].

The majority of families in the study were impacted by the recession. Given the dependence of some African families on social welfare, the very significant effect of the recession on this group is not surprising. Most such families in our study reported reductions in social welfare with consequences for paying rent and utility bills. In contrast, the reported impact of the recession for EU-10 families was comparable to the impact on Irish families. EU-10 fathers were however more likely to have lost their jobs, reflecting their employment in the sectors most impacted on by the recession.

Despite the impact of the recession, rates of depression at Wave 2 and self-rating of health as poor/fair were relatively low. The impact of the recession on these outcomes was only seen in Irish fathers and not EU-10 or African fathers. This is similar to the findings of Simona-Moussa and Ravazzini [6] who reported that the effects of financial crises were seen in objective indicators such as income poverty and not in subjective indicators such as wellbeing for some potentially vulnerable groups. They suggested that these groups already had experience of coping with financial instability and uncertainty. In our study, the pattern of under-employment of African fathers was evident prior to the recession and this pattern persisted throughout the recession and recovery [21].

Grosser et al. [28] highlighted the potential of population cohort studies such as birth cohorts for investigating health inequalities and the importance of enrolling diverse and potentially disadvantaged groups in these studies. The follow-up of these diverse groups, however, may be challenging. Transient accommodation and moving for work may have a more significant impact on follow-up for migrant families. We identified lower rates of follow-up for migrant fathers, particularly EU-10 fathers, at Wave 3. Employment in this group contracted by over 26% nationally between 2007 and 2012 and emigration increased steadily throughout the crisis [15]. Further research on attrition rates of migrants in population cohort studies is needed and the development of effective strategies for recruitment, follow-up and analysis.

Limitations of this study include the relatively low number of African fathers, though the proportion is higher than the national rate (3% vs. 1.4% from the most recent Census). Only families from which both mothers and fathers were interviewed were included in this study. To account for the impact of the recession at Wave 2, only families who participated in both Wave 1 and Wave 2 were included in the analysis. The impact of the recession on the household was reported by the mother as primary caregiver. Rates of depression and self-rated health as poor/fair were low and there is uncertainty in the estimates with wide confidence intervals. Longer term analysis is limited by differences in follow-up rates across groups. Our focus was on first-generation migrant fathers given the relatively recent inward migration into Ireland and only 1 % of the population estimated to be second-generation migrants with both parents born abroad [17].

## Conclusion

Understanding the relationship between economic conditions and health outcomes is complex and may be related to multiple dimensions of socio-economic advantage, disadvantage and structural inequalities. African families were already more likely to be disadvantaged prior to the recession and that pattern persisted during the recession. Further research on attrition rates of migrants in population cohort studies is needed and the development of effective strategies for recruitment, follow-up and analysis.

## Data Availability

Data can be accessed on application to the Irish Social Science Data Archive at www.ucd.ie/issda.

## Footnotes

1 Asylum seeking fathers were included if the mother of the child was eligible for Child Benefit
